# Sex differences in sepsis outcomes across the lifespan: a population-based cohort study in Germany

**DOI:** 10.1186/s13054-025-05657-4

**Published:** 2025-09-26

**Authors:** Norman Rose, Islam Agrama, Irit Nachtigall, Mathias W. Pletz, Jenny Rosendahl, Ha-Yeun Chung, Christina E. Zielinski, Diana Dudziak, Melissa Spoden, Patrik Dröge, Stefan Hagel, Carolin Fleischmann-Struzek

**Affiliations:** 1 Institute of Infectious Diseases and Infection Control, Jena University Hospital, Stoystraße 3, 07743 Jena, Germany; 2 Centre for Sepsis Control and Care, Jena University Hospital, Jena, Germany; 3 Translational Research, Education and Cooperation at Vivantes Netzwerk für Gesundheit, GmbH and Medical School Berlin, Berlin, Germany; 4 Institute of Psychosocial Medicine, Psychotherapy and Psychooncology, Jena University Hospital, Jena, Germany; 5 Section Translational Neuroimmunology, Department of Neurology, Jena University Hospital, Jena, Germany; 6 Department of Infection Immunology, Institute of Microbiology, Leibniz Institute for Natural Product Research and Infection Biology, Friedrich Schiller University, Jena, Germany; 7 Institute of Immunology, Jena University Hospital, Jena, Germany; 8 AOK Research Institute (WIdO), Berlin, Germany

**Keywords:** Sepsis, Sex, Long-term outcome, Post-Sepsis-Syndrome, Mortality

## Abstract

**Importance:**

Sepsis is a major global health concern influenced by both biological sex and socially constructed gender roles, which can affect disease susceptibility, progression, treatment and outcomes. Evidence on sex-specific differences in sepsis often lacks age-specific analysis, despite known interactions between sex, age, and immune function.

**Objective:**

We aimed to investigate age-dependent associations between sex and mortality as well as long-term outcomes among sepsis survivors after hospitalization.

**Design, setting, and participants:**

This retrospective, population-based cohort study based on nationwide health claims data from 2009 to 2017 of 23.0 million beneficiaries of a large German health insurance provider. Patients aged 15 years and older with incident hospital-treated sepsis identified by ICD-10-GM codes in 2013 to 2014 were included.

**Exposures:**

Female and male sex.

**Main outcomes and measures:**

Differences in 12-months mortality, medical, psychological and cognitive diagnoses, as well as dependency on nursing care by sex and age were analyzed using generalized additive models including sex*age interaction effects. We report average marginal effects (AME) for sex and age as estimates of the adjusted marginal increase or decrease of the event rate of outcomes.

**Results:**

We included 159,684 sepsis patients in 2013/2014, of which 75,809 (47.5%) were female and 83,875 (52.5%) were male. The average marginal hospital and 12-months mortality over the observed age distribution was AME = − 2.8% (95% CI, − 3.2%, − 2.3%, *P* < .001) and AME = − 5.4% (95% CI, − 5.9%, − 4.9%, *P* < .001) lower in females, respectively. Significant female survival benefits were predominantly found beyond age 44 (hospital mortality) and age 47 (12-months mortality). Females were also less often affected by cognitive impairments, but more often experienced psychological and physical impairments as well as nursing care dependency with differential associations observable across the lifespan.

**Conclusion and relevance:**

Sepsis long-term outcomes appear to be influenced by a complex interaction between age and sex. While our study focuses on these factors, it is important to acknowledge that observed associations cannot be attributed to biological sex alone, as numerous additional factors - directly or indirectly related to sex- may also contribute. These findings underscore the importance of incorporating sex-specific considerations into sepsis care and post-acute support strategies to improve long-term outcomes.

## Introduction

Sepsis, which is caused by a dysregulated immune response to an infection [1], affects an estimated 50 million people annually and is associated with approximately 20% of deaths worldwide [2]. Similar to other diseases, there is growing understanding that sex and gender influence the development, presentation and outcome of sepsis. In this regard, sex refers to the biological differences between males and females, including chromosomes, hormones, and reproductive anatomy. These differences often result in sexual dimorphism—distinct physiological and anatomical characteristics between the sexes that can affect how diseases manifest and progress. In contrast, gender refers to the socially constructed roles, behaviors, identities, and expectations associated with being male, female, or non-binary [3].

In sepsis, sexual dimorphism includes, on the one hand, variations in the innate immune response due to genetic and epigenetic differences between women and men. On the other hand, sex hormones have been shown to interact with and modulate innate and adaptive immune responses in a complex manner [4, 5]. Furthermore, gender-specific aspects may impact prevention and health seeking behaviors, as well as the health care delivered in sepsis [6].

While male sex has long been considered a risk factor for the development of infection [7] and sepsis [8], a first analysis of sepsis incidence in the Global Burden of Disease framework found a higher age-adjusted sepsis incidence in women (717/100,000 population) than in men (643/100,000 population) in 2017 [2]. With regard to sepsis outcomes, previous studies found inconclusive results on the association between sex and sepsis hospital mortality, although most of the evidence was rated as low to very low certainty [9–11]. In common causative infections of sepsis, female survival benefits were found, e.g. in Staphylococcus aureus bacteremia [12] and severe pneumonia [13]. Concerning long-term outcomes [14], which are referred to as Post-Sepsis-Syndrome [15], evidence is scarce. A retrospective cohort study among Australian ICU patients observed lower three-year-survival rates among male patients [16]. Sex-related disparities in rehospitalizations after sepsis were described in a prospective cohort study including 264,678 adult ICU-treated sepsis patients [17], with male sepsis patients exhibiting higher age-adjusted rehospitalization rates than female sepsis patients.

One reason for the partly conflicting evidence on sex-specific outcome associations may be the fact that most studies consider outcomes across the lifespan. Such a summative analysis may not adequately reflect the interplay between age and sex, e.g. in terms of hormonal changes and sex-specific aging processes of the immune system [18]. To this end, a recent prospective multicenter study conducted by the Korean Sepsis Alliance found an age-dependent, non-linear association between sex and sepsis mortality. The study observed higher sepsis-related hospital mortality in females aged 19 to 50 years, while mortality rates were higher in males thereafter [19]. Given these findings and existing gaps in data on age-dependent sex disparities in sepsis long-term outcomes, this study aims to investigate differential associations between sex and mortality as well as Post-Sepsis-Syndrome impairments across the lifespan in a population-based cohort study based on nationwide health claims data in Germany.

## Materials and methods

The study forms part of the SEPFROK project, which was pre-registered (German Clinical Trials Registry ID: DRKS00016340) and approved by the Institutional Review Board of the Jena University Hospital (2019-1282-Daten). Reporting follows the Strengthening the Reporting of Observational Studies in Epidemiology reporting guideline.

### Database

We have conducted a population-based cohort study based on the nationwide health claims data from eleven legally independent German health insurance funds “AOK – Die Gesundheitskasse” from the years 2009 to 2017, which covers around one third of the German population [20]. The data included a complete record of hospitalizations, outpatient visits, nursing care dependency, sick leave days and vital status. The requirement for informed consent was waived as all data were deidentified. The use of data from 2009 to 2017 was determined by the design and timing of the SEPFROK study launched in 2019. Due to delays in data availability and processing, 2013–2014 represented the latest feasible inclusion period that still allowed for sufficient longitudinal outcome assessment period of three years (2025−2017) of the initial SEPFROK study.

### Identification of sepsis patients

Sepsis patients were identified among AOK-insured individuals older than 15 years based on explicit ICD-10-codes for sepsis coded as primary or secondary hospital discharge diagnoses in 2013–2014 [21, 22]. During that time frame, these codes were defined according to the sepsis-1/2 definition [21, 22], comprising patients with sepsis with and without organ dysfunction and septic shock. We excluded patients who were hospitalized with sepsis in the 24 months prior to hospital admission, and beneficiaries not consecutively enrolled in the insurance for five years prior and 36 months after sepsis hospitalization or until death. In the observation period, the first sepsis hospitalization was denoted as index hospitalization.

### Outcomes

We assessed all-cause in-hospital mortality among all sepsis patients. Additionally, we investigated the following long-term outcomes in the 12-months post-discharge among hospital survivors: all-cause mortality, medical, cognitive and psychological diagnoses, and long-term nursing care dependency. For all diagnoses, ICD-10-based definitions were developed based on literature review and expert consultation [14]. Medical diagnoses included: respiratory, cardiovascular, cerebrovascular, renal, hepatic, metabolic, urogenital and neuromuscular/musculoskeletal diagnoses, sensory disorders, anemia, fatigue, decubitus ulcer, pain, multidrug-resistant infections, complications of the tracheostomy and impairments of nutrition. Psychological diagnoses comprised depression, anxiety, PTSD, sleeping disorders and substance abuse. Cognitive diagnoses included mild to severe cognitive impairment as a single diagnosis. Nursing care dependency which was defined according to German graded care level system, ranging from Grade 1: “Little impairment of independence” to “Grade 5: “Hardship cases”, or nursing home residence.

### Statistical analyses

We analyzed the stochastic dependencies of the selected outcomes on age and sex with respect to our research question. We used generalized additive models (GAM) for statistical analyses for two reasons. First, age ranged between 16 and 107 years in our sample cohort. The stochastic relationship between any outcome and age over the life course (e.g., the regression curve) is rarely linear or follows a known functional form. Second, we assumed an interaction effect between age and sex regarding the outcomes. Hence, we expected sex-specific regression curves that differ between females and males. GAMs allows for simultaneous estimation of sub-group specific smoothing splines for metric variables (i.e., age) and for testing interaction effects between factors and metric variables (i.e., sex and age) in the model [23].

In order to test the Null hypothesis of no sex × age interaction, we used generalized likelihood ratio test (GLRT), which compares the full model *M*_*F*_ that allows for sex × age interaction and the restricted model without the interaction [23]. The model equation of *M*_*F*_ for a binary outcome variable *Y* is $\:g\left[P\left(Y=1\:|\:age,\:\:sex\right)\right]=\alpha\:+\beta\:\bullet\:sex+s\left(age\:\right|\:sex)$. α denotes the intercept and g[] the logit link function. The interaction was taken into account by estimating separate penalized cubic spline regression curves for females and males within the same model, as expressed by the conditional smooth component $\:s\left(age\:\right|\:sex)$. The parameter $\:\beta\:$ represents the conditional group difference between both sexes given the predictor *age* at the logit metric. We report unadjusted *p*-values of the tests of the Null hypothesis H_0_: $\:\beta\:$ = 0 as well as the p-value of the Wald-type *χ*^2^-tests of the Null hypotheses H_0_: *s*(*Age* | *sex = female*) = 0 and H_0_: *s*(*Age* | *sex = male*) = 0 [24]. For a better illustration, the results are also presented graphically, including (a) the estimated regression curves for both sex groups, (b) the estimated slopes of these regression curves depending on age, (c) the estimated conditional probability differences between males and females, and (d) the differences in the estimated sex-specific slopes of the regression curves. The slope of the regression curve at particular age is interpreted as the instantaneous change in the event rate (e.g., the increase or decrease in the mortality at this age). Differences in the sex-specific slopes of the regression curves depending on age provide more detailed information regarding the question how sex and age interact. We also report average marginal effects (AME) for sex and age as estimates of the adjusted marginal increase or decrease of the event rate [25, 26].

The exclusive consideration of hospital mortality has been criticized as insufficient for describing mortality in patients with bloodstream infections [27]. To obtain a more comprehensive picture of mortality for both sexes after surviving sepsis, we used methods of survival analysis. Kaplan-Meier curves were estimated for sepsis survivors with 95% confidence intervals. Additionally, direct nonparametric smoothed estimate of the hazard function based on B-splines and generalized linear mixed models (GLMM) are obtained [28]. The Kaplan-Meier curves and hazard curves were estimated for females and males separately within the four age strata; age < 40 years, 41 ≤ age ≤ 65 years, 66 ≤ age ≤ 80 years, and age > 80 years. Differences in the post-sepsis mortality between females and males over the first year were tested using the log-rank test and the differences in the restricted mean survival time (Diff_RMST_) within each age strata [29–31]. The restricted mean survival time (RMST) is the average survival time up to a specific time point, which is the day 360 after hospital discharge in our study [32].

All statistical analyses were conducted using the free software R [33] with r packages mgcv and marginal effects [23, 34]. For survival analyses in R the additional r package survival, bshazard and surv2RM were used [28, 35, 36].

## Results

**Fig. 1:**
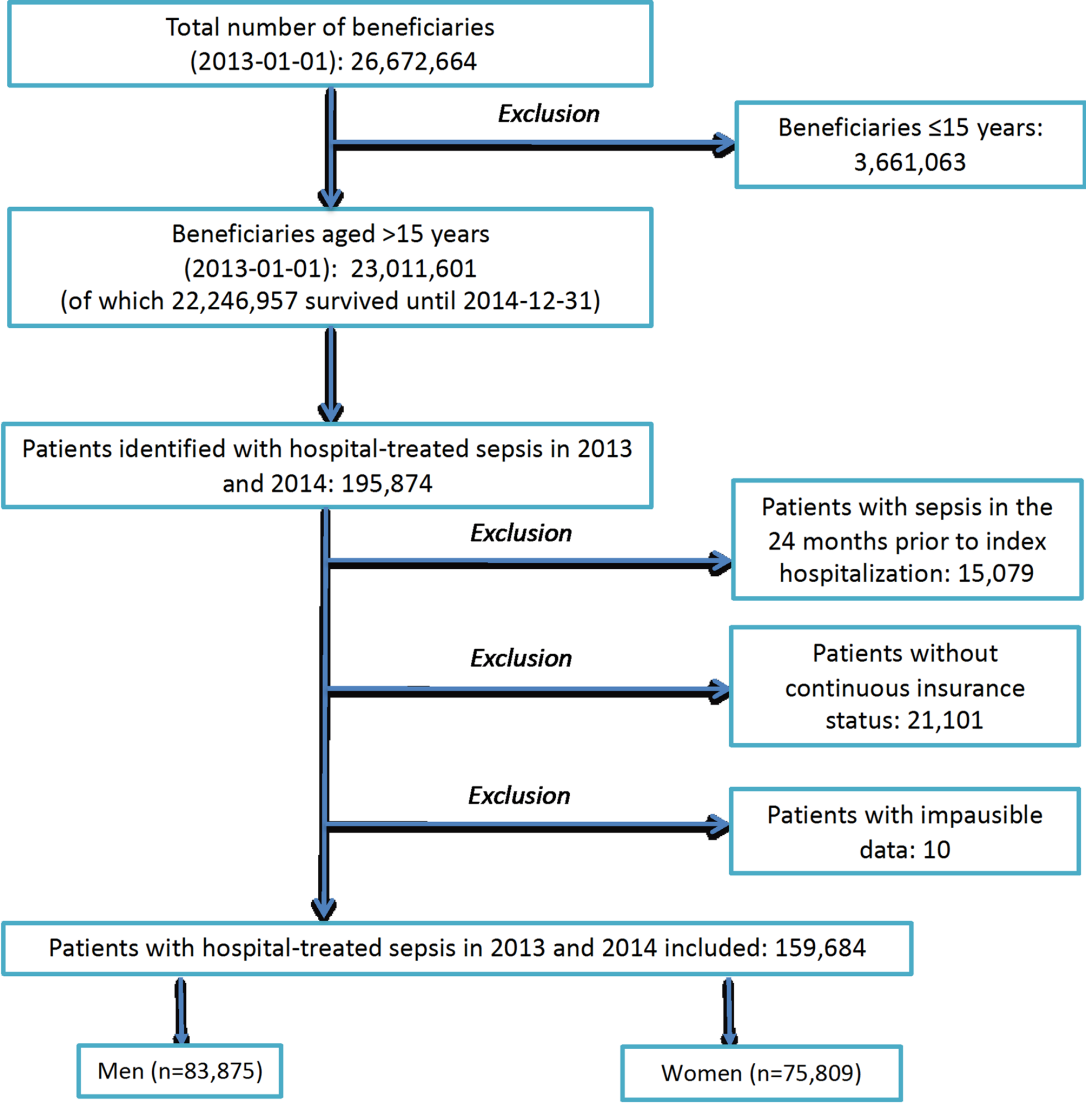
Flow of patient inclusion

Among 23.0 million beneficiaries, we identified 159,684 sepsis patients in 2013/2014 (Fig. 1), of which 75,809 (47.5%) were female and 83,875 (52.5%) were male. Female patients were older than male patients (mean age 75.8 vs. 72.0 years, *P* <.001, Table 1) and had a higher degree of care dependency pre-sepsis (45.42% vs. 33.42%, *P* <.001). The spectrum of comorbidities differed between both sexes, with a lower prevalence of cancer (22.2% vs. 29.3%, *P* <.001), liver disease (15.7% vs. 20.4%, *P* <.001), chronic pulmonary disease (31.1% vs. 36.5%, *P* <.001), and drug or alcohol abuse (5.2% vs. 12.9%, *P* <.001) in female compared to male patients. Women exhibited higher prevalence rates for congestive heart failure (40.7% vs. 38.1%, *P* <.001), hypertension (83.9% vs. 79.5%, *P* <.001), and obesity (27.7% vs. 22.6%, *P* <.001). During the index hospitalization, women were less likely to be treated with severe sepsis (41.6% vs. 45.8%, *P* <.001) and septic shock (11.9% vs. 13.8%, *P* <.001). Differences were also observed in the focus of infections, with women being less frequently affected respiratory tract infections (33.2% vs. 43.3%, *P* <.001), but they had a higher prevalence of urogenital tract infections (37.0% vs. 24.9%, *P* <.001). Regarding treatment intensity, female sepsis patients were less often treated at the ICU (29.7% vs. 37.9%, *P* <.001), received mechanical ventilation less frequently (21.1% vs. 28.8%, *P* <.001), and had a lower rate of renal replacement therapy (8.2% vs. 12.2%, *P* <.001) during their hospitalization.

**Table 1:** Demographics, baseline characteristics, and clinical features of female and male patients with sepsis

Demographics & baseline characteristics	Female (*N* = 75809)	Male (*N* = 83875)	*p* ^a^
Age in years, mean (SD); median (IQR)	75.8 (13.0); 78.0 (14)	72.0 (12.4); 75.0 (16)	< 0.001
Nursing care grade			< 0.001
No nursing care grade	1.9 [1.3, 2.5]	2.3 [1.7, 3.0]	
Nursing care grade 2	37.9 [37.3, 38.5]	34.8 [34.2, 35.5]	
Nursing care grade 3	30.5 [29.9, 31.1]	30.6 [30.0, 31.3]	
Nursing care grade 4	20.5 [19.9, 21.0]	22.5 [21.9, 23.1]	
Nursing care grade 5	9.2 [8.7, 9.8]	9.7 [9.1, 10.4]	
Nursing home residence (%)	14.6 [14.4, 14.9]	9.0 [8.8, 9.2]	< 0.001
**Comorbidities**			
Congestive heart failure, %	40.7 [40.3, 41.0]	38.1 [37.8, 38.5]	< 0.001
Diabetes, %	46.6 [46.3, 47.0]	46.1 [45.8, 46.4]	0.036
Hypertension, %	83.9 [83.6, 84.1]	79.5 [79.2, 79.8]	< 0.001
Chronic pulmonary disease, %	31.1 [30.8, 31.5]	36.5 [36.2, 36.8]	< 0.001
Renal disease, %	35.3 [34.9, 35.6]	34.9 [34.6, 35.2]	0.141
Cancer, %	22.2 [21.9, 22.5]	29.3 [28.9, 29.6]	< 0.001
Dementia, %	22.7 [22.4, 23.0]	17.2 [16.9, 17.4]	< 0.001
Drug and alcohol abuse, %	5.2 [5.0, 5.3]	12.9 [12.7, 13.2]	< 0.001
Liver disease, %	15.7 [15.4, 15.9]	20.4 [20.1, 20.6]	< 0.001
Obesity, %	27.7 [27.4, 28.0]	22.6 [22.3, 22.9]	< 0.001
**Index sepsis and hospitalization**			
Focus of infection			
Respiratory, %	33.2 [32.9, 33.5]	43.3 [42.9, 43.6]	< 0.001
Abdominal, %	16.4 [16.2, 16.7]	15.5 [15.3, 15.8]	< 0.001
Wound/soft tissue, %	6.1 [6.0, 6.3]	6.2 [6.0, 6.3]	0.858
Urogenital, %	37.0 [36.6, 37.3]	24.9 [24.6, 25.1]	< 0.001
Central nervous system, %	0.9 [0.8, 0.9]	0.9 [0.9, 1.0]	0.159
Vascular system, %	3.3 [3.2, 3.4]	3.7 [3.6, 3.8]	< 0.001
Device-related, %	6.4 [6.2, 6.5]	8.1 [7.9, 8.3]	< 0.001
Pregnancy-associated, %	0.1 [0.1, 0.2]	0.0 [0.0, 0.0]	< 0.001
Hospital-acquired infection, %	18.5 [18.3, 18.8]	23.1 [22.8, 23.4]	< 0.001
**Organ dysfunctions**			
Severe sepsis, %	41.6 [41.3, 42.0]	45.8 [45.4, 46.1]	< 0.001
Septic shock, %	11.9 [11.7, 12.2]	13.8 [13.5, 14.0]	< 0.001
Respiratory failure, %	38.0 [37.6, 38.3]	44.3 [44.0, 44.7]	< 0.001
Encephalopathy, %	14.1 [13.9, 14.3]	16.9 [16.7, 17.2]	< 0.001
Renal failure, %	28.9 [28.6, 29.2]	30.5 [30.2, 30.8]	< 0.001
Metabolic dysfunction, %	8.5 [8.3, 8.7]	10.0 [9.8, 10.2]	< 0.001
Hepatic failure, %	2.6 [2.5, 2.8]	3.4 [3.3, 3.5]	< 0.001
Coagulopathy, %	8.9 [8.7, 9.1]	10.8 [10.6, 11.0]	< 0.001
**Treatment characteristics**			
Hospital length of stay, mean (SD), median (IQR)	19.3 (19.5); 13.0 (16)	21.7 (21.8); 15.0 (18)	< 0.001
Surgical treatment, %	28.3 [28.0, 28.6]	34.4 [34.0, 34.7]	< 0.001
Intensive care treatment, %	29.7 [29.4, 30.0]	37.9 [37.6, 38.3]	< 0.001
Mechanical ventilation, %	21.1 [20.8, 21.4]	28.8 [28.4, 29.1]	< 0.001
Dialysis, %	8.2 [8.0, 8.4]	12.2 [12.0, 12.4]	< 0.001

^a^ *p* value of the *χ*^2^-Test of the Null hypothesis that females and males do not differ in the distribution of the characteristics (categorical variables), or the *p* value of the Welch Test of the Null hypothesis that females and males have equal population means (metric variables)

### Acute and long-term all-cause mortality

**Fig. 2:**
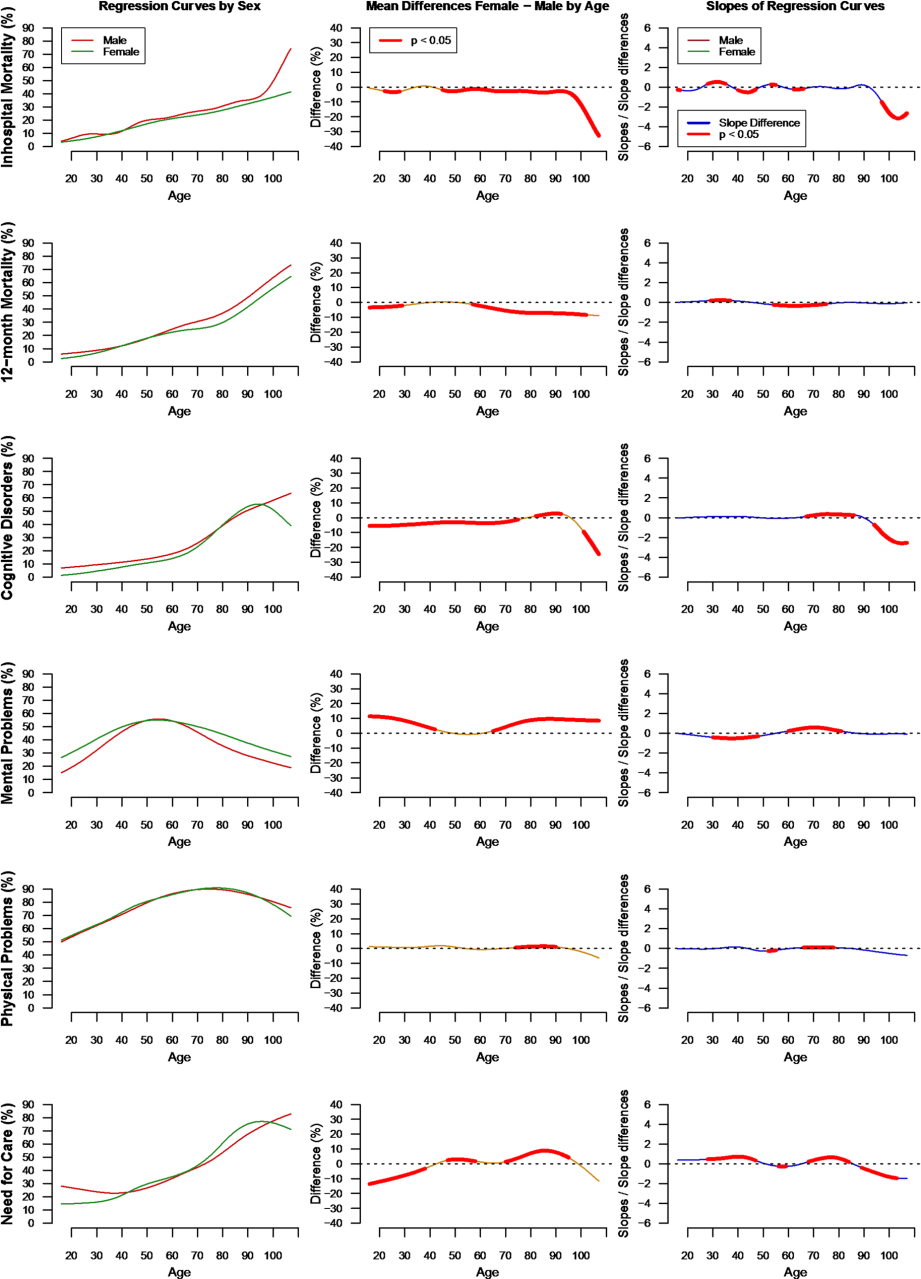
Estimated sex-specific regression curves of the six outcomes on age (left). Difference between age-specific proportions of the outcome between female and male sepsis survivors are shown in the middle column, and differences in the slopes of sex-specific regression curves (differential increase or decrease) at each age are shown in the right column

**Fig. 3:**
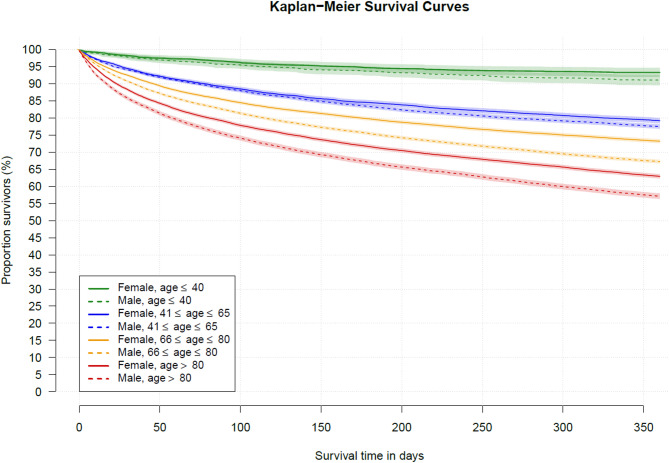
Estimated sex-specific Kaplan-Meier survival curves for the four age strata (see legend)

**Fig. 4:**
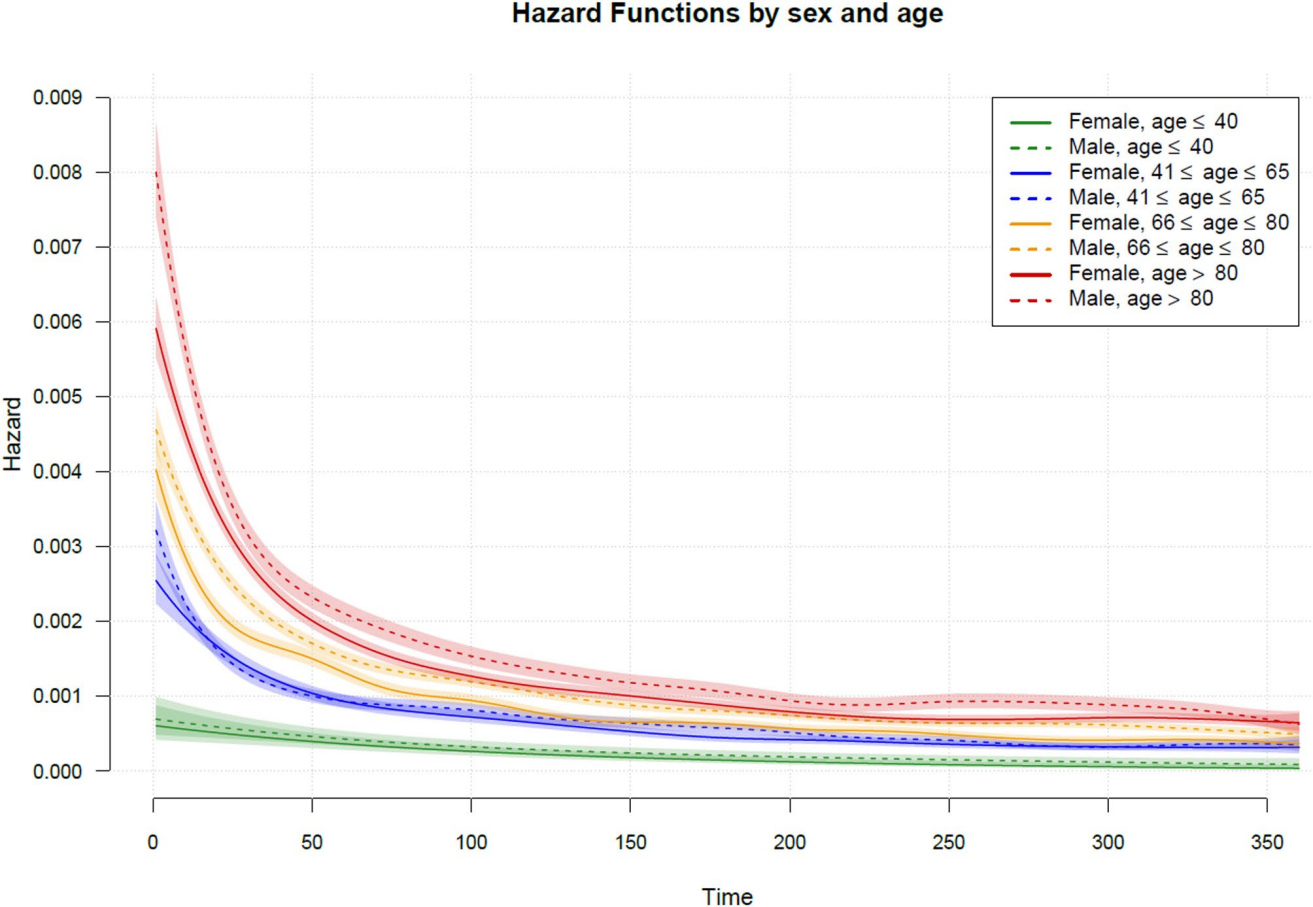
Estimated sex-specific Hazard curves for the four age strata (see legend)

In-hospital mortality was 26.4% in female and 27.6% in male patients. At 12 months, 31.9% of female and 29.3% of male hospital survivors had died. The average marginal hospital and 12-months mortality over the observed age distribution was AME = −2.8% (95% CI, −3.2%, −2.3%, *P* <.001) and AME = −5.4% (95% CI, −5.9%, −4.9%, *P* <.001) lower in females, respectively. Across the lifespan, the in-hospital and 12-months mortality rates increased steadily with age in both sexes (Fig. 2). Significant female survival benefits were predominantly found beyond 44 years (hospital mortality) and 47 years (12-months mortality). A significant sex-age interaction was observable for the 12-months mortality (*χ*^2^(7.16) = 54.86, *P* <.001), which was mainly due to a stronger increase in 12-months mortality in middle-aged male (age 54–75) compared to female patients (Fig. 2). The age- and sex-specific Kaplan-Meier curves show that the survival benefit for female sepsis survivors is the lowest among the youngest age group (i.e., age ≤ 40) and the largest among the oldest age group (i.e., age > 80, see Fig. 3). Although the log-rank test shows a statistically significant higher risk to die for males than females in all age strata (Table 2), the difference in RMST were not statistically significant in the youngest age strata (Diff_RMST_ = 4.3, 95% CI, −1.1, 9.8, *P* = 0.118). However, the RMST was significantly lower among males than females in all older age strata (see Table 3). The estimated Hazard functions in Fig. 4 reveal that the risk to die was the highest immediately after hospital discharge for both sexes and for all age groups. Sex disparities in the hazard rates were also most pronounced among older sepsis survivors closely after hospital discharge. The risk of death was the highest among older patients. The hazard functions show a sharp initial decline until day 40–50 and little change from day 150 onwards.

**Table 2:** Absolute and relative frequencies (percentages) of deceased among male and female sepsis survivors for each age groups, within the first year after hospital discharge

Age (years)	Females		Males		Log. Rank Test	
	Deceased/*N*	Percentage (95% CI)	Deceased/*N*	Percentage (95% CI)	χ^2^ (df = 1)	*p*
< 40	89/1321	6.7 (5.5,8.2)	119/1328	9.0 (7.5,10.6)	4,435	0.035
41 ≤ Age ≤ 65	2061/9889	20.8 (20.1,21.7)	3600/15,971	22.5 (21.9,23.2)	9,910	0.002
66 ≤ Age ≤ 80	6056/22,582	26.8 (26.2,27.4)	9581/29,205	32.8 (32.3,33.3)	210,110	< 0.001
> 80	8162/21,989	37.1 (36.5,37.8)	6097/14,222	42.9 (42.1,43.7)	124,378	< 0.001

**Table 3:** Restricted mean survival times (RMST) with 95% CI for male and female sepsis survivors within the four age strata, and the two-sided test of the RMST differences (Diff_RMST_) between males and females for each strata

Age (years)	RMST in days		Diff_RMST_		
	Females	Males	Days	CI-95	*P* ^a^
< 40	342.9 (339.3,346.6)	338.6 (334.6,342.6)	4.3	(−1.1, 9.8)	0.118
41 ≤ Age ≤ 65	308.7 (306.5,310.9)	305.0 (303.2,306.7)	3.7	(0.9, 6.5)	0.009
66 ≤ Age ≤ 80	292.8 (291.2,294.4)	278.5 (277.0,279.9)	14.3	(12.1,16.5)	0.000
> 80	265.9 (264.0,267.7)	250.1 (247.8,252.5)	15.7	(12.7,18.7)	0.000

^a^ Unadjusted p-value of the test of the Nullhypothesis that female and male sepsis survivors do not differ in their RMST (i.e., Diff_RMST_ = 0) within the first 360 days after hospital discharge

### Prevalence of diagnoses

Table 4 shows the prevalence of diagnoses post-sepsis in both sexes. The age-adjusted marginal prevalence of cognitive diagnoses until 12-months post-sepsis was slightly lower for females until the age of 75, with on average AME = 0.9% (95% CI, 0.3%, 1.14%, *P* =.001) less females than males with cognitive impairments after sepsis. The steady increase in the prevalence accelerated substantially between 60 and 90 years and was more pronounced in females compared to males (GLR-Test for sex*age interaction: *χ*^2^(6.61) = 109.27, *P* <.001). With regard to psychological diagnoses, we observed an inverted u-shaped relationship between age and the prevalence of psychological disorders for both sexes, with the highest prevalence at age 54 in females (55.0%, 95% CI, 53.8%, 56.2%) and age 55 in males (55.8%, 95% CI, 54.7%, 56.8%). Hence, the prevalence of psychological impairments increased until this peak and declined afterwards (see Fig. 2D). The increase as well as the decline was weaker in females in the age groups 30–48 and 60–82 years respectively (GLR-Test for sex*age interaction: *χ*^2^(6.46) = 170.09, *P* <.001). No significant differences in the prevalence between female and male sepsis survivors were observed between 43 and 64 years. However, outside this age range, a higher prevalence was found in females than males, with on average AME = 6% (95% CI, 5.4%, 6.6%, *P* <.001) more psychological diagnoses across the lifespan.

**Table 4:** Relative frequencies (percentage) of sepsis outcomes for female and male patients

Outcomes of sepsis	Female	Male	*p* ^c^
Inhospital Mortality^a^	26.4 [26.1, 26.7]	27.6 [27.3, 27.9]	< 0.001
12-months Mortality^b^	29.3 [29.0, 29.7]	31.9 [31.6, 32.3]	< 0.001
Cognitive Diagnoses^b^	29.3 [29.0, 29.7]	31.9 [31.6, 32.3]	< 0.001
Mental Diagnoses^b^	33.9 [33.6, 34.3]	30.2 [29.8, 30.6]	< 0.001
Physical Diagnoses^b^	45.5 [45.1, 45.9]	42.1 [41.7, 42.5]	< 0.001
Chronic nursing care dependency^b^	87.9 [87.7, 88.2]	87.2 [87.0, 87.5]	< 0.001

^a^ refers to all *N* = 159,684 hospital treated sepsis patients (females: *N* = 75,809; males: *N* = 83,875)

^b^ refers only to the *N* = 116,507 survivors of the hospital treated sepsis (females: *N* = 55,781; males: *N* = 60,726)

^c^ *p* value of the *χ*^2^-Test of the Null hypothesis that women and men do not differ in the probability of occurrence of the outcome

The relationship between the occurrence of physical impairments after sepsis and age was very similar for females and males. A slightly higher prevalence was found in women between 74 and 90 years, with on average AME = 0.7% (95% CI 0.3%, 1.1%, *P* <.001) more women than men with a physical diagnosis. Prevalences increased over time with a significant sex*age interaction (GLR-Test: *χ*^2^(7.02) = 18.41, *P* =.010, Fig. 2) and plateaued around the age of 70 years.

### Chronic nursing care dependency

In line with the increasing numbers of sepsis survivors with physical and cognitive impairments, the prevalence of nursing care dependency increased continuously with age (Fig. 2). However, a significant age*sex interaction effect was found (GLR-Test: *χ*^2^(6.58) = 156.51, *P* <.001) due to a stronger increase among females between 28 and 47 and 68 and 84 years. This explains the higher prevalence of nursing care dependency among females (AME = 4.4%, 95% CI, 3.8%, 4.9%, *P* <.001) despite the lower initial prevalence in younger females aged < 38 years. However, this trend shifted towards a higher prevalence in female patients found beyond 47 years.

## Discussion

Examining sex-related disparities in sepsis outcomes in a nationwide population-based sepsis cohort, we found a 2.8% lower in-hospital and 5.4% lower 12-months sepsis all-cause mortality rate in females. They also were less often affected by cognitive impairments, but suffered more frequently from psychological and physical impairments as well as nursing care dependency. However, these sex-related differences vary significantly depending on age. For example, female survival advantages were observed from 44 to 47 years onwards, stressing the importance to consider age-sex interactions in studies assessing sex-outcome associations in sepsis.

Previous research was inconclusive with regard to sex-related survival differences, although the majority of long-term follow-up studies suggests survival benefits in women for up to three years [10, 11, 16, 17]. These studies used heterogenous study designs and were mostly conducted among ICU populations in high- and middle-income countries, which may limit the comparability to our data [10]. In our study, we observed both, lower in-hospital and 12-months all-cause mortality in females, driven by survival benefits beyond 44–47 years. This finding may arise from various reasons. First, it may mirror sex-specific differences in the immune response during clinical sepsis, e.g. by sex-specific expression of pro- and anti-inflammatory cytokines. In experimental studies, women showed increased expression of anti-inflammatory mediators, contributing to better control of the inflammatory response. Furthermore, estrogens were found to have immunomodulatory effects and to enhance the activity of immune cells, while testosterone can dampen the immune response, particularly cell-mediated immunity [37]. The second x chromosome also makes a difference, it harbors a high density of genes involved in both innate and adaptive immunity. Most parts of the additional part are inactivated but up to 23% can escape inactivation and influence immunological events contributing to stronger immune responses [38]. Second, differences may arise from differential patient characteristics, such as sex-specific pattern of comorbidities, with higher rates of cancer, liver and pulmonary diseases found in men. These differences may be related to differences in health behavior [39, 40] and biological differences, i.e. due to genetic and epigenetic mechanisms, as well as the effect of sex hormones [41]. Contrary, female patients were less often affected by septic shock and suffered more frequently from genitourinary sepsis, which was found to be associated with lower mortality rates compared to other sites of infection in sepsis [42]. They also were less frequently treated in the ICU and received mechanical ventilation, which may indicate a lower severity of disease. Third, other mechanisms, such as differential responses to antibiotic or fluid treatment due to differences in the metabolic or cardiovascular system as well as varying baseline hemodynamics and responses to stress, may also play a role, but are yet insufficiently understood [43].

The observed age-dependent differences in all-cause mortality are in line with a previous study from Korea which also found a female survival benefit only in higher age groups [19]. Similarly, a matched cohort study based on the OutcomeRea database observed that hospital mortality was significantly lower in women (OR, 0.69; 95% CI, 0.52, 0.93) > 50 years, while no differences were found in patients  ≤50 years [44]. Also in COVID-19, a lower mortality was found in elderly women [45]. One explanation lies in the interplay between hormonal and chromosomal immune influences. While estrogen enhances humoral immunity, its decline after menopause leads to shifts in inflammatory and immune responses [5]. However, unlike hormone-dependent immunity, the T-cell response, which is partially regulated by X-linked immune genes [46], remains more robust in females throughout life [47]. This sustained advantage in adaptive immunity could contribute to the lower long-term mortality seen in older women. In contrast, men experience a progressive decline in T-cell function with age [48, 49], exacerbating their sepsis-related mortality risk. Treatment disparities and sex-based physiological differences may further shape this difference. Women often receive less aggressive intensive care [50–52], which could be harmful in younger years but protective in older age. This complex interplay underlines the importance of considering not only sex, but also age-sex-interactions in future research.

Another important finding is that the pattern of long-term impairments also differed by sex and age. A higher prevalence of cognitive impairment was found in males, particularly in younger age groups. This aligns with preclinical studies demonstrating significant differences in gene expression, with an upregulation of apoptosis-related and inflammatory response genes and a downregulation of genes related to neurogenesis and gliogenesis in male mice [53, 54]. Such changes are likely associated with the fact that male sex is linked to an increased delay in cognitive recovery from sepsis [55]. The exact cause of these alterations remains unclear. However, it appears that several metabolic and systemic factors, such as the presence of steroid hormones, sex chromosome-related gene expression, differential cell death, and immune pathways, may contribute to poor neurocognitive outcomes in male mice [56]. From a clinical perspective, it is well established that male patients have a higher incidence of delirium during sepsis, which is recognized as the primary risk factor for developing cognitive impairment in sepsis survivors [57]. Psychological impairments, contrary, affected 6% more female patients in the 12-months post-sepsis. A similar sex gap was also observable in studies among the general population, with females showing a higher incidence rate of any psychiatric disorder than males at age 15–59 and 70–99 years [58]. Besides differences in neurological processes [59], this may also mirror gender-biased diagnosis and treatment of mental health impairments, which became evident e.g. for depression [60]. It has been postulated that on the one hand, clinician-related factors lead to a possible overdiagnosis of mental health impairments in women and underdiagnosis in men [61]. On the other hand, also mental health screening tools could favor the expression of female over male depression symptoms and therefore lead to biased estimates [62]. Furthermore, the health seeking behavior for mental health impairments was found to be higher in women than in men [63], also after prolonged ICU stay [64], which impacts the prevalence detected in health claims data. With regard to physical impairments, a slightly higher prevalence was found in younger females, and female survivors had higher rates of nursing care dependency, which may be driven also by higher rates of baseline care dependency. Moreover, the higher mortality of males in the observation period may also contribute to higher nursing care dependency among females.

Our study has several limitations. First, our study used health claims data that include only a binary classification of patient sex (“male” or “female”) as recorded in insurance documents. Information on gender identity was not available. Therefore, our analyses are limited to biological sex as officially registered and do not capture the potentially important influence of gender identity and gender-related factors such as role expectations, behavior, or discrimination in healthcare settings. Second, although our data includes a complete record of patient diagnoses, mortality and nursing care grades, we are only able to detect so-called administrative prevalences, which are defined as proportions of individuals identified as having a specific condition based on diagnosis codes or records within administrative healthcare data. Being recorded as such requires the patient to seek medical treatment and the physician to diagnose and code a disease correctly. This may lead to an underestimation of disease prevalence, the extent of which is difficult to estimate. This also applies for the case identification of sepsis patients, which was found to be of limited validity in claims data compared to the gold standard of clinical sepsis diagnosis in patient charts. This, particularly, missed patients with lower case severity [65]. Furthermore, this limitation also affects the detection of long-term sequelae, particularly in the domains of mental health and cognitive impairment. Previous analyses from the SEPFROK project showed that the prevalence of diseases such as PTSD is considerably higher when assessed through prospective patient-reported outcome measures [66, 67], than when derived from administrative data. Similarly, more patients report subjective cognitive impairments than having a diagnosis. This suggests that our study likely underestimates the true burden of psychological and subclinical cognitive impairments among sepsis survivors. In order to capture broader aspects of functional health not dependent on diagnostic coding, we additionally included new nursing care dependency as a complementary outcome. This indicator is based on standardized assessments by the Medical Service of the Health Insurance Funds and reflects functional decline requiring informal or formal care support, independently of physician-assigned diagnoses. Third, the present study is based on health claims data from the AOK health insurance funds, which cover approximately one third of the German population. While the AOK-insured population is broadly representative with regard to age and sex distribution, previous studies have indicated differences compared to the overall population. Specifically, AOK beneficiaries tend to have a slightly lower socioeconomic status and a higher burden of chronic diseases [68]. These differences may influence healthcare utilization and outcomes. Furthermore, AOK has stronger representation in certain regions, particularly in eastern and southern Germany, which may introduce some geographic variation [68]. This may limit the generalizability of the results. Fourth, although we did not perform time-to-event analyses for physical, cognitive, mental health outcomes and nursing care need, as we lack concrete information on the date of diagnosis, competing risks such as death may still influence the interpretation of our results. Specifically, differences in outcome proportions between female and male sepsis survivors may in part reflect differential mortality during follow-up. One example may be that male survivors had higher 12-month mortality rates, which may have reduced their likelihood of being diagnosed with outcomes that typically develop over time, such as mental health impairments. While this does not bias the estimated proportions, it may contribute to observed group differences and therefore should be considered in the interpretation of findings. Fifth, acute mortality was operationalized as hospital mortality, as the exact timing of sepsis onset could not be determined from the available health claims data. Moreover, we were unable to determine the cause of death, as health claims data do not contain validated cause-of-death information. Therefore, we report all-cause mortality, which remains a relevant endpoint for outcome studies in sepsis survivors. Further studies using clinical datasets with detailed timing of sepsis onset and validated cause-of-death information are needed to explore sex-specific differences in mortality with greater precision. Sixth, the GAMs describe the nonlinear stochastic relationship between the explanatory variables (i.e., sex and age) regarding the selected outcomes. The associations between the variables cannot be interpreted as causal effects per se and our analyses are limited in that we did not analyze the causal pathways that finally led to the observed differences between female and male sepsis survivors at different ages. We acknowledge that a variety of additional factors can influence the occurrence of the considered outcomes. However, we did not include such variables in our statistical models because they do not meet the definition of a confounder. A confounder is defined as a common cause of the focal variable (e.g., treatment variable) and the outcome variables [69]. Since none of the available variables in our dataset temporally precedes biological sex, they cannot be considered confounders requiring adjustment.

## Conclusion

In conclusion, sepsis outcomes appear to be shaped by a complex interplay between age and sex. While our study focuses on these factors, it is important to note that observed associations cannot be attributed to biological sex alone, as numerous additional factors, directly or indirectly related to sex, may also influence outcomes. A deeper understanding of the mechanisms and consequences of this interplay could help inform sex- and age-sensitive approaches in sepsis treatment, ultimately improving care for both sexes.

## Data Availability

The authors confirm that the data used in this study cannot be made available in the article, the supplemental files, or in a public repository due to German data protection laws (Bundesdatenschutzgesetz [BDSG]). Therefore, they are stored on a secure drive in the AOK Research Institute to facilitate replication of the results. Generally, access to data of statutory health insurance funds for research purposes is possible only under the conditions defined in German social law (SGB V §287). Requests for data access can be sent as a formal proposal specifying the recipient and purpose of the data transfer to the appropriate data protection agency. Access to the data used in this study can only be provided to external parties under the conditions of the cooperation contract of this research project and after written approval by the sickness fund. For assistance in obtaining access to the data, please contact [wido@wido.bv.aok.de](mailto: wido@wido.bv.aok.de).
